# Reliability of health-related quality-of-life assessments made by older adults and significant others for health states of increasing cognitive impairment

**DOI:** 10.1186/s12955-016-0579-3

**Published:** 2017-01-07

**Authors:** Gina Bravo, Modou Sene, Marcel Arcand

**Affiliations:** 1 Department of Community Health Sciences, Faculty of Medicine and Health Sciences, Université de Sherbrooke, Sherbrooke, QC Canada; 2 Research Centre on Aging, University Institute of Geriatrics of Sherbrooke, 1036 South Belvedere Street, Sherbrooke, QC Canada J1H 4C4; 3 Department of Family Medicine, Faculty of Medicine and Health Sciences, Université de Sherbrooke, Sherbrooke, QC Canada

**Keywords:** Health-related quality of life, Elderly, Proxy, Consistency, Agreement, Cognitive impairment, Generalizability theory

## Abstract

**Background:**

Older adults are encouraged by many organizations to engage in advance care planning in the event of decisional incapacity. Planning for future health care often involves anticipating health-related quality of life (HRQoL) in states of reduced cognitive functioning. No study has yet examined whether anticipated HRQoL is stable over time. The accuracy with which significant others can predict how an older adult envisions HRQoL in a future state of cognitive impairment is also unknown. We investigated the extent to which health-related quality-of-life ratings made by older adults and designated proxies for health states of increasing cognitive impairment are consistent over time and agree with each other.

**Methods:**

Results are based on HRQoL ratings made on a 5-point Likert scale by 235 community-based elder-proxy dyads on three occasions. Ratings were obtained for the older adult’s current health state as well as under the assumption that he/she had a mild to moderate stroke, incurable brain cancer or severe dementia. Data were analyzed using both traditional approaches (e.g., intraclass correlation coefficients, Bland-Altman plots) and the theory of generalizability.

**Results:**

We found ratings to be reasonably consistent over time and in good agreement within dyads, even more so as implied cognitive functioning worsened. Across health states, ratings over time or within elder-proxy dyads were no more than one category apart in over 87% of cases. Using the theory of generalizability, we further found that, of the two facets investigated, rater had a greater influence on score variability than occasion.

**Conclusions:**

These findings underscore the importance of discussing health-related quality-of-life issues during advance care planning and involving designated proxies in the discussion to enhance their understanding of the role that HRQoL should play in actual decision-making situations. Medical decision-making may be influenced by healthcare providers’ and family members’ assessments of an incapacitated patient’s health-related quality of life, in addition to that of the designated proxy. Future studies should investigate whether these two groups of individuals share the views of the patient and the designated proxy on anticipated HRQoL.

**Electronic supplementary material:**

The online version of this article (doi:10.1186/s12955-016-0579-3) contains supplementary material, which is available to authorized users.

## Background

Advance care planning is a process by which a person thinks about and communicates his/her healthcare preferences in the event of decisional incapacity to significant others. People formulate preferences taking several factors into account, including the expected benefits and harms of treatment options. Other factors often at play are one’s current health-related quality of life (HRQoL) and appraisal of HRQoL in future health states that would impair decision-making capacity [1–4]. For instance, Bravo et al. [4] have recently reported that older adults who rated HRQoL as unbearable should they be severely demented were 2.7 times more likely to opt for comfort care only rather than life-prolonging care when compared to older adults who provided a more positive rating of HRQoL in severe dementia. It is widely agreed that the individuals themselves are the best source of information regarding internalized constructs such as health-related quality of life and preferences for care in various health conditions [5–9]. Evidence suggests that a large majority of people with mild to moderate cognitive impairment, and some with severe dementia, can provide meaningful insight into their quality of life and preferences for care [7, 10, 11]. However, a considerable proportion of patients who require medical decision-making are incapable of providing such insight [12, 13]. According to Torke et al. [13], 68% of hospitalized adults aged 65 and older faced at least one major decision in the first 48 h of hospitalization. Surrogate decision makers were involved in these decisions for nearly half of the patients. All decisions were made by a surrogate in 23% of cases. Decisional incapacity may have resulted from a sudden health event (e.g., stroke) that caused major communication difficulties (e.g., aphasia) or from reaching an advanced stage of a disease (e.g., dementia) that progressively erodes cognitive functioning. In these circumstances, the medical team will commonly involve family members in the decision-making process, with the purpose of jointly establishing the best course of action for the incapacitated patient [2, 14].

Prior research indicates that, like patients who retain decision-making capacity, family members take health-related quality of life into consideration when making decisions on behalf of an incapacitated loved one [2–4, 15, 16]. Their judgment of the quality of life of a cognitively impaired patient may thus have significant implications for the type of treatment and level of care provided to the patient [5, 17–20]. Hence, it is important to know the extent to which significant others can accurately assess patients’ quality of life in health states that impair cognitive functioning. Paradoxically, the accuracy of substituted judgment cannot be examined for patients who most need family members’ involvement in decision making, i.e., those who are decisionally incapacitated [18, 19]. As an alternative, researchers have compared family assessments to those of persons with various degrees of cognitive impairment yet still capable of self-report or, in fewer instances, to those made by patients after they have regained capacity [16]. Equivalence of family ratings and self-reports has been studied across a range of conditions, including stroke, cancer, and dementia [e.g., 11, 20–22]. Results vary across studies, due in part to differences in measurement instruments, analytical approaches, and the population under investigation. However, most researchers have reported suboptimal agreement between patient and family assessments, indicating somewhat different perspectives on the patient’s quality of life. Agreement was typically found to be stronger for more overt aspects of quality of life (e.g., physical functioning) and weaker for internally experienced psychosocial domains. In cases of disagreement, close relatives tended to underestimate the patient’s health-related quality of life. Some studies [5, 6] have further suggested a U-shaped relationship between patient-family agreements and patient’s health status, with larger discrepancies occurring more frequently among patients who are slightly or moderately impaired, and less frequently among those who are doing either very poorly or very well.

For the most part, the findings briefly summarized above were based on health-related quality-of-life assessments made for a patient’s current health situation. We know of no studies that have compared family ratings to self-reports of HRQoL made in the context of hypothetical health states as typically contemplated when engaging in advance care planning. We recently conducted a randomized trial in which older adults and significant others provided such ratings. By analyzing these data it is possible to determine the level of agreement between older adults and relatives on anticipated HRQoL. As pointed out by Sneeuw et al. [6], “high levels of patient-proxy agreement cannot reasonably be expected when either one provides ratings with compromised reliability.” To our knowledge, the reliability of HRQoL ratings made for hypothetical health states has not been reported. In the aforementioned trial, participants provided HRQoL ratings on three separate occasions. Estimating the reliability of these ratings provides a frame of reference for interpreting agreement indices. This paper reports findings from secondary analyses of these data aimed at investigating the extent to which health-related quality-of-life ratings made by older adults and significant others for health states of increasing cognitive impairment (1) are consistent over time and (2) agree with each other.

## Methods

### Sample and study design

The current analyses were based on data obtained from 235 elder-proxy dyads enrolled in a randomized trial promoting advance care planning [23]. The proxy was defined as the person the older adult would choose to make healthcare decisions on his/her behalf should the need arise. Eligible elders were French-speaking, community-dwelling adults aged 70 or older who were cognitively intact and had a proxy willing to co-participate in the trial. Potential elderly participants were randomly chosen from the administrative database of the Quebec universal health insurance plan. Selected individuals were sent a letter informing them of the study, and called one week later to assess their eligibility and willingness to enroll. The call included administering an investigator-designed 3-item memory test to screen out older adults who were likely unable to engage actively in advance care planning. The items asked the reason for the call (which was described in the letter they had received a week prior), the current date and the individual’s telephone number. Those deemed eligible who were interested in enrolling were requested to identify a proxy who we then contacted to assess his/her willingness to participate.

Following the baseline interview described below, elder-proxy dyads were randomly assigned to the experimental or control group. Experimental dyads attended three monthly activities aimed at motivating older adults to communicate their preferences for care to their proxies should they lose the capacity to make their own decisions. Control counterparts attended three monthly activities aimed at promoting healthy behaviors. The advance care planning intervention did not aim at modifying participants’ appraisal of health-related quality of life.

### Health-related quality-of-life assessments

The baseline interview (T_0_) was conducted at the authors’ research center by two specially trained senior nurses. Older adults and proxies were interviewed simultaneously, but in separate rooms so that their answers would not be contaminated. The nurses began the interview by asking participants to sign the consent form and provide some demographic information about themselves. Next, older adults were asked to rate their health-related quality of life in their current health state and again for three hypothetical states of increasing cognitive impairment: mild to moderate stroke, incurable brain cancer, and severe dementia. Nurses were provided with written material describing these states in lay terms to standardize the study participant task. Five response options were provided for rating HRQoL: *excellent*, *good*, *acceptable*, *poor*, and *unbearable*, coded from 1 to 5. Proxies were asked to provide ratings for the same four states, using the same response scale. The proxy question was identical to that of the older adult except for referring to the older adult rather than to the proxy personally. Proxies were explicitly instructed to answer from the older adult’s perspective, i.e., to rate health-related quality of life in each of the four states as they thought the older adult would (substituted judgment), rather than providing their own perspective on the older adult’s current quality of life and on what they believed the older adult’s quality of life would be like should he/she experience each of the three hypothetical health states. The interviews were repeated three months after the baseline (T_1_, i.e., shortly after the dyad’s last scheduled activity) and again 6 months later (T_2_). At these later two time points, older adults and proxies were interviewed by the same nurses who had interviewed them at baseline.

### Statistical analysis

Using SAS Proc MIXED [24] to test the group-by-time interaction, no effect of the advance care planning intervention on HRQoL was discerned among either older adults or proxies (cf. Additional File 1). The same conclusion was reached when Friedman’s nonparametric test was used instead to investigate within-group changes in ratings over time (*p*-values ranging from 0.184 to 0.952 among older adults, and from 0.107 to 0.434 among proxies). Consequently, data from the experimental and control dyads were pooled for subsequent analyses, ignoring the group to which dyads were initially allocated.

For each of the four health states, we conducted two complementary sets of analyses to study consistency of health-related quality-of-life ratings over time and level of agreement within elder-proxy dyads. The first set followed a more traditional approach while the second was based on the theory of generalizability introduced by Cronbach and his associates in 1972 [25].

#### Traditional approach

First, raw data were scrutinized by computing the percentages of (i) identical responses (either over time or within dyads), and responses that differed (ii) by only one category, and (iii) by more than one category. Second, intraclass correlation coefficients (ICCs) and their 95% confidence intervals [26] were derived from two-way models to quantify rating consistency over time and agreement within dyads. Subjects and occasions were both treated as random effects while raters were treated as fixed. As an aid to interpreting the ICCs, we applied the following standards often used for reliability indices: values above 0.75 were considered indicative of excellent agreement, between 0.40 and 0.75 as fair to good, and below 0.40 as poor agreement [27]. The level of rater agreement was further examined graphically by plotting the differences in older adults’ and proxies’ ratings against the mean of their ratings [28, 29]. Bland-Altman plots were also generated to visualize the consistency of ratings over occasions, this time plotting the standard deviation of ratings against the mean [30].

#### Generalizability theory

As opposed to classical test theory, which can only estimate a single source of measurement error at a time, generalizability theory enables one to estimate the relative magnitudes of multiple sources of variation (called *facets*) simultaneously in a single analysis [31]. Facets are akin to factors in analysis of variance and represent any set of conditions under which measurements can be carried out [32]. Generalizability theory uses analysis of variance approaches to decompose a person’s score into an effect for persons (called the *object of measurement*), an effect for each facet, and an effect for each of their combinations. In the present context, two facets were under investigation: rater (older adults versus proxies) and occasion (three repeated measures over time). The greater the inconsistencies between the two raters, the more hazardous it would be to generalize from a proxy rating to the universe of interest. Similarly, to the extent that ratings are inconsistent across occasions, generalization from the ratings collected on one occasion to the universe of ratings across all occasions of interest is hazardous. Facets can be random or fixed. A facet is random if the conditions under which measures were taken could be exchanged for any other same-size set of possible conditions. It is fixed when the conditions of the facet used in the study exhaust all conditions to which one wants to generalize [33]. Here, rater is a fixed facet while occasion is random. Health state was not considered a facet because averaging ratings across health states is of no clinical interest. Rather, variance components were estimated separately for each health state, paralleling the traditional approach described above. Variance estimates were obtained using the G1 program developed for SAS by Mushquash and O’Connor [34].

## Results

Baseline characteristics of the 235 older adults and proxies enrolled in the trial are fully described elsewhere [35, 36]. In brief, older adults were 77.6 years of age on average (SD = 4.6) and 46% were female. Proxies were slightly younger (mean age = 70.3, SD = 10.5) and predominantly female (69.8%). Both groups had an average of 13 years of schooling (SD = 4.8 and 4.2 for older adults and proxies, respectively). In two-thirds of cases (66.8%) the proxy was the spouse of the older adult; in 19.1%, his or her child. In the remaining cases (14.1%), the proxy was another family member (e.g., sibling, niece) or a friend. In no case was the proxy a healthcare provider. Nearly half of older adults (44.7%) rated their health as fair-to-poor. Of the 235 dyads initially enrolled, 188 provided health-related quality-of-life ratings at T_1_, and 185 at T_2_. Two dyads that provided ratings at T_2_ were absent at T_1_. As a result, 183 dyads provided ratings on all three occasions.

### Traditional approach to investigating reliability

**Fig. 1:**
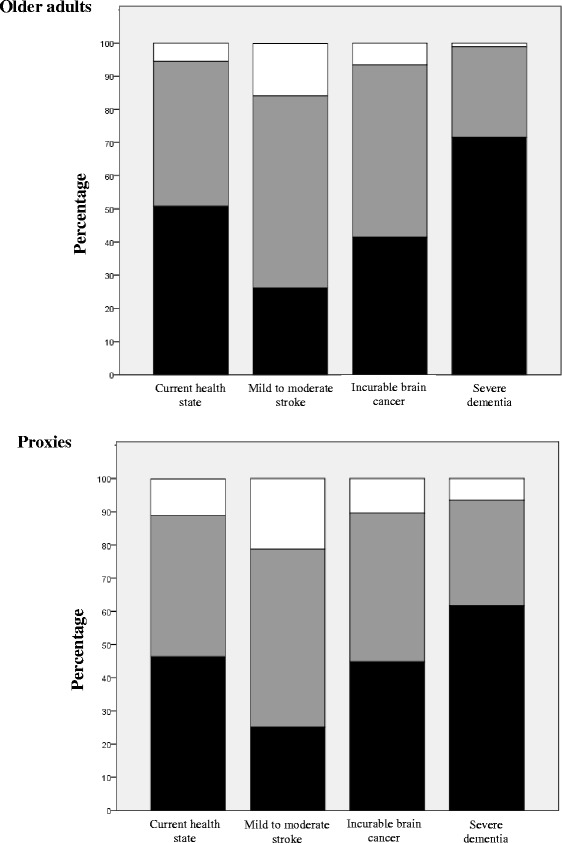
Consistency of health-related quality-of-life ratings over time, among older adults and proxies, for each health state (*n* = 183). ■: Identical responses; : Responses differ by at most one category; □: At least two responses differ by more than one category

Separately for older adults and proxies, Fig. 1 shows the percentage of individuals who gave identical health-related quality-of-life ratings over the three time points, of those whose ratings differed by at most one category, and of those whose ratings were more than one category apart at least once. Combining health states, 92.8% of older adults gave ratings that differed by no more than one category while this was the case for 87.7% of proxies. Among both older adults and proxies, greater variability in ratings was found for the mild to moderate stroke scenario and less for the severe dementia state.

**Fig. 2:**
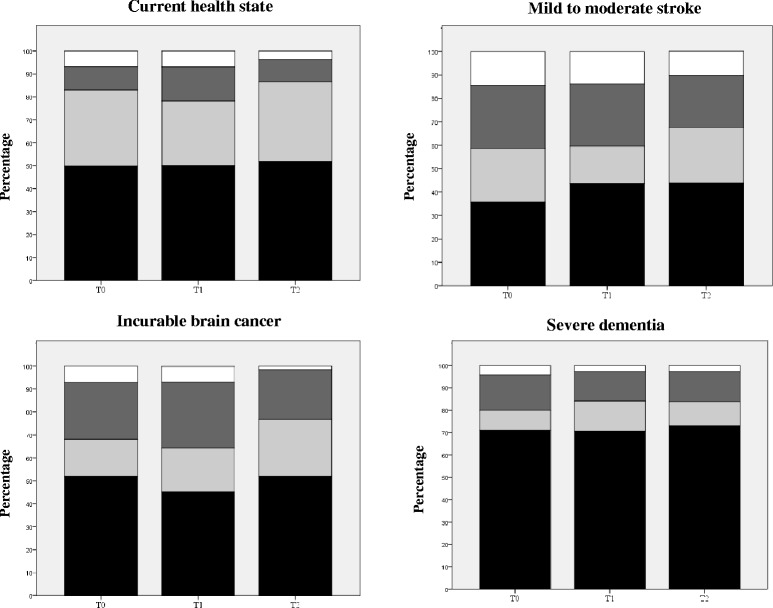
Agreement between older adults and proxies on health-related quality-of-life ratings, for each health state and time point (*n* = 235 at T_0_, 188 at T_1_, and 185 at T_2_). ■: Identical responses;  = Proxies overestimate by one category;  = Proxies underestimate by one category; □ : Ratings differ by more than one category

Figure 2 shows the extent to which older adults and proxies agreed on their ratings, for each health state and at each time point. Overall, agreement is quite high if one considers a distance of one category between ratings as acceptable. Across time points and health states, over 87% of dyads’ ratings were one category or less apart. Seventy percent of dyads gave identical ratings for the state in which proxies would be most needed, i.e., severe dementia. When proxy ratings disagreed with those given by the older adult, proxies tended to rate HRQoL more positively, with the exception of the current health state, where the opposite was more prevalent.

A rather different picture emerges from the intraclass correlation coefficients (ICCs) reported in Table 1. Coefficients are poor, except in a few instances involving the current health state, where most are fair. ICCs are similar between older adults and proxies (confidence intervals overlap), except for the severe dementia state, where older adults are more consistent over time than proxies. ICC point estimates vary somewhat over the three occasions. However, because confidence intervals overlap for all four health states, differences in ICCs are not statistically significant.

**Table 1:** Intraclass correlation coefficients and 95% confidence intervals for the consistency of health-related quality-of-life ratings over time and level of agreement between raters, according to health state

	Current health state	Mild to moderate stroke	Incurable brain cancer	Severe dementia
Consistency over time^a^				
Older adults	0.55	0.41	0.39	0.49
	(0.47, 0.63)	(0.32, 0.50)	(0.30, 0.48)	(0.40, 0.57)
Proxies	0.55	0.27	0.28	0.20
	(0.46, 0.62)	(0.16, 0.37)	(0.14, 0.41)	(0.11, 0.30)
Agreement between raters^b^				
T_0_	0.44	0.16	0.19	0.12
	(0.33, 0.53)	(0.04, 0.28)	(0.06, 0.31)	(−0.01, 0.24)
T_1_	0.38	0.19	0.01	0.02
	(0.25, 0.50)	(0.05, 0.33)	(−0.13, 0.15)	(−0.13, 0.16)
T_2_	0.51	0.26	0.23	0.07
	(0.40, 0.61)	(0.12, 0.39)	(0.09, 0.36)	(−0.07, 0.22)

^a^Results derived from a two-way random-effects model

^b^Results derived from a two-way mixed-effects model

**Fig. 3:**
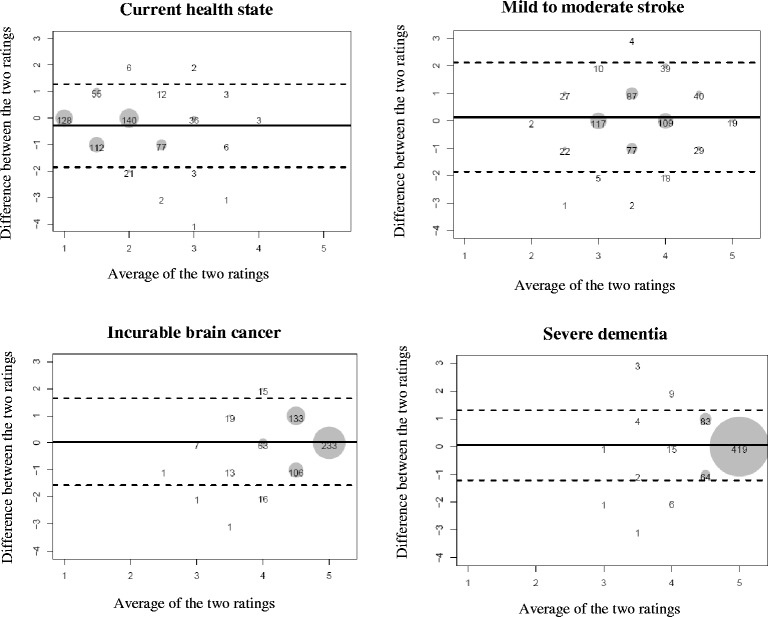
Bland-Altman plots, combining ratings over the three time points (*n* = 608). The solid horizontal line is drawn at the mean of the differences in ratings ($\overline{d}$) within dyads, thus providing an estimate of the amount of overall bias. The two dashed lines are 95% limits of agreement defined as $\overline{d}$ ± 1.96 *s*_*d*_ where *s*_*d*_ is the standard deviation of the differences corrected for clustering [29]. These lines delimit the range within which 95% of the differences lie. Scores below the zero difference line indicate that the proxy provided a higher rating than the older adult (i.e., poorer health-related quality of life), and conversely for scores above that line

Bland-Altman plots are shown in Fig. 3 for each health state, combining the three time points. Plots depict similar patterns over time, hence our decision to combine ratings across time in four single plots. As expected, average ratings tend to shift to the right of the plot as cognitive impairment increases. Bias is small for all four scenarios: slightly negative for the current health state (−0.28, implying that proxies rated the older adult’s current health-related quality of life more negatively) and slightly positive for the three other states (from 0.04 to 0.13). Most differences are close to the zero difference line, and increasingly so as cognitive functioning worsens. The limits of agreement are farther apart in the mild to moderate stroke scenario (implying more discordant ratings), and closer for the severe dementia state.

**Fig. 4:**
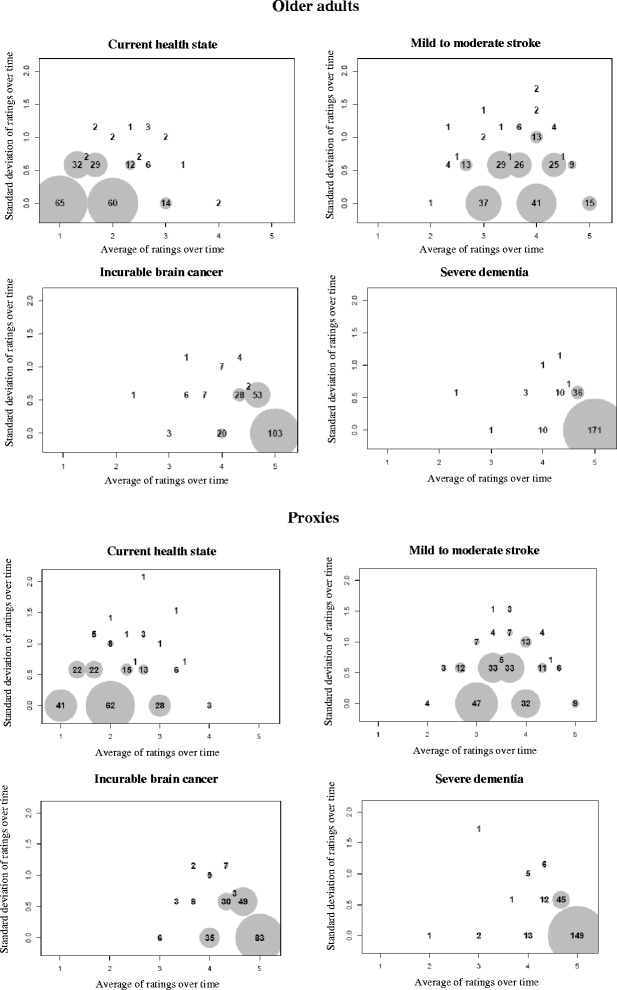
Scatter plots of the standard deviations of ratings over time against their means, by health state, for (**a**) older adults and (**b**) proxies (*n* = 235)

Complementing Fig. 3, the last figure focuses on the consistency of ratings over the three occasions, separately for older adults and proxies. The standard deviation of repeated ratings is below 0.5 for most older adults (Fig. 4a). Very few ratings are above this cut-off for the hypothetical state of severe dementia, while outliers are more numerous under the mild to moderate stroke scenario. The same patterns are observed among proxies (Fig. 4b). However, for this latter group, standard deviations are larger for all four health states, suggesting that proxies were somewhat less consistent over time in how they rated the older adult’s health-related quality of life.

### Using generalizability theory to investigate reliability

Estimates of variance components are reported in Table 2, separately for each health state. Consistent with Figs. 3 and 4, the estimated person variance decreases (from 0.205 to 0.025) when moving from the current health state to a state of severe dementia. This means that, averaging over raters and occasions, variability in HRQoL ratings becomes smaller as implied cognitive impairment increases. The other large estimated variance components concern the rater facet more than the occasion facet. Variability attributable to occasions is negligible for all four health states, indicating that ratings are relatively constant over the three interviews when averaged across persons and raters. Moreover, the two interactions involving occasions collectively account for less than 5% of the variance. Variability due to raters is also relatively small, except for the current health state, where it accounts for 6.5% of the overall variance in ratings. The person by rater interaction accounts for 13.7% (mild to moderate stroke) to 21.3% (severe dementia) of the total variability, reflecting different relative standings of persons across raters. Lastly, the large residuals reflect a three-way interaction between persons, raters, and occasions and/or other sources of error not systematically incorporated in the study. These two possibilities cannot be disentangled.

**Table 2:** Sources of variation in health-related quality-of-life ratings estimated from a two-facet fully-crossed design, according to health state (*n* = 183)

Source of variation	Estimated variance component	Percent of total variability
Current health state		
Person	0.205	34.3
Rater	0.039	6.5
Occasion	0.000^a^	0.0
Person x Rater	0.100	16.8
Person x Occasion	0.029	4.8
Rater x Occasion	0.000^a^	0.0
Residual	0.224	37.6
Mild to moderate stroke		
Person	0.133	20.4
Rater	0.007	1.1
Occasion	0.004	0.7
Person x Rater	0.089	13.7
Person x Occasion	0.001	0.2
Rater x Occasion	0.000^a^	0.0
Residual	0.418	64.0
Incurable brain cancer		
Person	0.067	16.4
Rater	0.000^a^	0.0
Occasion	0.000^a^	0.1
Person x Rater	0.066	15.9
Person x Occasion	0.000^a^	0.0
Rater x Occasion	0.000^a^	0.1
Residual	0.277	67.5
Severe dementia		
Person	0.025	11.1
Rater	0.000^a^	0.0
Occasion	0.000^a^	0.0
Person x Rater	0.049	21.3
Person x Occasion	0.000^a^	0.0
Rater x Occasion	0.003	1.2
Residual	0.152	66.5

^a^Negative variances set to zero as recommended by Cronbach et al. [25]

## Discussion

High-quality substitute decision-making requires adequate knowledge of the incapacitated patient’s goals of care. These, in turn, are partially based on health-related quality-of-life considerations. Using data initially gathered for another purpose, we investigated the stability of HRQoL ratings obtained over a 9-month period from older adults and proxies for health states of increasing cognitive impairment. We also studied the extent to which older adults and proxies agreed on their ratings across these health states.

### Consistency over time

Overall, we found that ratings were relatively stable over time, especially when participants were asked to envision themselves or their relatives in a state of severe dementia. For each of the four health states, stability was slightly greater among older adults rating their own health-related quality of life than among proxies trying to guess the older adult’s assessment. This reflects the greater difficulty of determining what would constitute an acceptable HRQoL for another person. A number of factors could be at play (e.g., the number of aspects that were taken into account when rating quality of life and how each was weighted). To study the consistency of a measure over time, one has to assume that the construct being measured is constant within the measurement period. Change may have occurred in the health state of some older adults over the 9 months during which they were involved in the study, with HRQoL ratings changing accordingly. This phenomenon would explain some of the variability over time in ratings observed for the current health state. For the three hypothetical states, it is reasonable to assume that the construct being measured—anticipated health-related quality of life—remained relatively constant over time. Figs. 1 and 4 clearly show that the stability of ratings increases with the degree of hypothetical cognitive impairment, among both older adults and proxies.

### Agreement between raters

We reach a similar conclusion regarding agreement. Proxies were relatively accurate in predicting older adults’ health-related quality of life under the four health scenarios. Moreover, the level of agreement was relatively constant over time (cf. Fig. 2). Figure 2 further shows a pattern similar to that seen in Fig. 1, i.e., agreement increasing with the implied severity of cognitive impairment. Higher agreement under the dementia scenario is an important finding, as this is a state for which there may be no alternative to substitute decision-making. Our conclusion that proxies are reasonably accurate in their predictions rests on the premise that ratings differing by no more than one category would be satisfactory for most purposes. Arguably, whether one rates health-related quality of life as *excellent* or *good* is unlikely to adversely affect medical decision-making for the patient. The same likely holds for rating HRQoL as *poor* or *unbearable*. This may not be true, however, of patients for which one rater judged HRQoL as *acceptable* while the other judged it as *poor* (still a difference of one category on our response scale). This situation occurred in 8.7% of the 608 pairs of ratings. Although relatively rare according to our data, such situations may affect the care provided to a patient. They should be investigated in future studies, with the objective of finding ways to decrease their occurrence even further. For instance, one could test whether an advance care planning intervention leads to fewer such discrepant ratings when quality of life is discussed at greater length in the presence of the proxy. Issues to discuss could include, for example, what health-related quality of life means to the participant, what makes it acceptable and less acceptable, and what role it should play in decision-making situations involving decisional incapacity. It would also be of interest to investigate patient-proxy agreement on anticipated HRQoL in states of decisional incapacity due to specific causes. For instance, stroke associated with severe aphasia could affect HRQoL differently than cognitive impairment. Lastly, future studies could expand the rater conditions to include healthcare professionals. Medical decision-making is influenced by many factors, including providers’ perception of a patient’s quality of life. Evidence is needed as to whether their perception coincides with that of patients and proxies. In practice, medical decision-making for an incapacitated patient will often involve more than one of the patient’s relatives. The extent to which relatives agree on anticipated HRQoL should also be investigated in the future.

### Methodological contributions

From a methodological point of view, this study highlights the value of combining approaches to study the reliability of measurements. The more traditional approach, although informative, makes it difficult to determine which of two factors (here, rater and occasion) has greater influence on score variability. By contrast, generalizability theory makes it clear that, in the present context, rater is more influential than occasion (cf. Table 2). The present study also highlights the limitations of the intraclass correlation coefficient (ICC) as an indicator of reliability. From Table 1, one might erroneously conclude that reliability tends to decrease as cognitive impairment increases. The decrease in ICCs as cognitive functioning worsens reflects a decrease in score variability rather than poorer consistency over time or agreement between raters. It is an artifact, as pointed out by Müller and Büttner [37]. ICCs are low in homogeneous populations. In the present context, one expects variability in health-related quality-of-life ratings to decrease as cognitive impairment increases, and hence ICCs to also decrease.

### Strengths and limitations

Strengths include the relatively large sample size, random selection of prospective elderly participants, clear instructions on how to select proxies, and standardization of the measurement process, both over time and between raters. A potential limitation is the administration of a short memory test over the telephone to exclude older adults who would likely be unable to engage actively in advance care planning (ACP). The test was not validated beforehand and hence may have excluded individuals who were, in fact, able to engage in ACP, thereby affecting the external validity of the study. For those who pass the screening test and were thus included in the study, the first encounter with the research nurse provided an additional opportunity to identify older adults who, despite passing the test, seemed too cognitively impaired to participate in the study. No older adult was excluded a posteriori on the basis of the nurse’s clinical judgment.

As is typical of this type of study, the hypothetical nature of the health contexts in which measurements were taken limits their generalizability to actual disease states. While health-related quality of life could be (and has been) studied among participants who had sustained a stroke or suffered from brain cancer [e.g., 8, 20, 21], this would not be possible in most adults with severe dementia. Another limitation is that, by necessity, current findings were derived from a single-item global measure of health-related quality of life and may not apply to comprehensive multi-item scales. HRQoL is a multidimensional construct most often measured with multiple items [38, 39]. Multi-item questionnaires are known to generate scores that are more reliable, precise and sensitive to change in clinical status than their single-item counterparts. Yet the scientific literature supports the usefulness of single items and their validity [40–43]. They have several advantages over multi-item questionnaires, including simplicity, ease of use and minimization of respondent burden. Moreover, the current paper provides evidence of the reliability of the single question we used to measure overall HRQoL. Lastly, in the clinical setting, health-related quality of life would most likely be explored using simple questions similar to the one we used.

## Conclusions

From this study, we conclude that health-related quality-of-life ratings made by older adults and designated proxies are reasonably consistent over time and in good agreement for most purposes. This is reassuring, as facilitators of advance care planning will often begin their intervention by asking participants to reflect on quality of life and to factor HRQoL considerations into their wishes for future health care. Efforts should nonetheless be undertaken to ensure greater consensus on anticipated HRQoL in states of decisional incapacity, given its known influence on goals of care.

## Additional file

Additional file 1. Health-related quality-of-life assessments across groups and time points, among older adults and proxies, according to health state. (DOCX 14 kb)
