# Supplementary material for: Reliability of health-related quality-of-life assessments made by older adults and significant others for health states of increasing cognitive impairment
Source: Health Qual Life Outcomes. 2017 Jan 7;15:4. doi: 10.1186/s12955-016-0579-3 (PMC5220615; doi:10.1186/s12955-016-0579-3)
Supplement: Additional file 1. — Health-related quality-of-life assessments across groups and time points, among older adults and proxies, according to health state. (DOCX 14 kb) [file 12955_2016_579_MOESM1_ESM.docx]

**Additional File 1.** Health-related quality-of-life assessments across groups and time points, among older adults and proxies, according to health state

| **Health state** | **Experimental Group** | | | *p*-value^b^ | **Control Group** | | | *p*-value^b^ | *p*-value^c^ |
| --- | --- | --- | --- | --- | --- | --- | --- | --- | --- |
|  | **T_0_** | **T_1_** | **T_2_** |  | **T_0_** | **T_1_** | **T_2_** |  |  |
|  | **Older adults** | | | | | | | | |
| Current health state | 1.62  (0.07)^a^ | 1.69  (0.07) | 1.63  (0.07) | 0.867 | 1.64  (0.07) | 1.69  (0.07) | 1.72  (0.07) | 0.310 | 0.639 |
| Mild to moderate stroke | 3.70  (0.09) | 3.62  (0.08) | 3.63  (0.08) | 0.688 | 3.63  (0.09) | 3.54  (0.09) | 3.74  (0.09) | 0.093 | 0.275 |
| Incurable brain cancer | 4.48  (0.07) | 4.55  (0.07) | 4.53  (0.07) | 0.710 | 4.53  (0.07) | 4.56  (0.06) | 4.58  (0.06) | 0.791 | 0.887 |
| Severe dementia | 4.85  (0.04) | 4.81  (0.05) | 4.82  (0.04) | 0.651 | 4.84  (0.04) | 4.80  (0.05) | 4.80  (0.05) | 0.545 | 0.981 |
|  | **Proxies** | | | | | | | | |
| Current health state | 1.93  (0.08) | 1.90  (0.08) | 1.83  (0.08) | 0.448 | 1.98  (0.08) | 1.94  (0.08) | 2.11  (0.09) | 0.088 | 0.055 |
| Mild to moderate stroke | 3.56  (0.08) | 3.43  (0.09) | 3.61  (0.07) | 0.193 | 3.48  (0.09) | 3.44  (0.08) | 3.60  (0.08) | 0.219 | 0.766 |
| Incurable brain cancer | 4.52  (0.07) | 4.44  (0.06) | 4.58  (0.06) | 0.184 | 4.50  (0.07) | 4.50  (0.07) | 4.60  (0.06) | 0.356 | 0.784 |
| Severe dementia | 4.71  (0.06) | 4.83  (0.04) | 4.79  (0.04) | 0.144 | 4.70  (0.07) | 4.78  (0.05) | 4.84  (0.04) | 0.116 | 0.471 |

^a^ Data reported are estimated marginal means with standard error in parentheses. Means vary from 1 to 5, with higher scores reflecting poorer health-related quality of life.

^b^ From testing within-group change in health-related quality-of-life ratings over time.

^c^ From testing the group-by-time interaction.
